# Essential Oil of *Cymbopogon citratus* on the Control of the *Curvularia* Leaf Spot Disease on Maize

**DOI:** 10.3390/medicines4030062

**Published:** 2017-08-20

**Authors:** Dalmarcia de Sousa Carlos Mourão, Talita Ferreira de Souza Pereira, Danival José de Souza, Aloísio Freitas Chagas Júnior, Mateus Sunti Dalcin, Ronice Alves Veloso, Evelynne Urzêdo Leão, Gil Rodrigues dos Santos

**Affiliations:** Federal University of Tocantins, Gurupi Campus, 77402-970 Gurupi, Brazil; dalmarciaadm@yahoo.com.br (D.S.C.M.); cupufer@gmail.com (T.F.S.P.); danival@uft.edu.br (D.J.S.); chagasjraf@uft.edu.br (A.F.C.J.); m2d@uft.edu.br (M.S.D.); ronicealves@hotmail.com (R.A.V.); evelynnegpi@hotmail.com (E.U.L.)

**Keywords:** botanical fungicide, lemongrass, *Zea mays*, preventive control, curative control

## Abstract

The *Curvularia* Leaf Spot is becoming more common due to the culture expansion and the low resistance of the cultivated genotypes in tropical regions. Thus, the objective was to evaluate the fungitoxicity of the essential oil of *Cymbopogon citratus* upon the phytopathogen *Curvularia lunata*, causative agent of the *Curvularia* Leaf Spot. There was realized pathogenicity tests of *C. lunata* in maize plants, phytotoxicity of the essential oil of *C. citratus* and gas chromatography attached, germination tests of the conidia, and of in vitro inhibition of *C. lunata*. Also, there were realized tests aiming at verifying the phytopathogen control in vivo. In the pathogenicity tests, there were verified symptoms of the disease in all of the suspensions tested on plants. It was observed that the essential oil concentrations of 7.5 µL mL^−1^ to 50 µL mL^−1^ were phytotoxic. The majoritarian chemical components of the essential oil of *C. citratus* were Geranial (41.46%) and Neral (32.43%). The concentrations of 5 and 7.5 µL mL^−1^ inhibited 100% of conidia germination. None of the concentrations evaluated effectively inhibited *C. lunata* mycelial growth in in vitro tests. In the preventive control, the concentration of 7.5 µL mL^−1^ was sufficient for the reduction of the progress of the disease, however the curative control was not efficient on the tested dosages.

## 1. Introduction

Even Brazil, occupying a highlighting position between the biggest world’s producers, has a productivity that is considered low when compared to other producer countries. In the state of Tocantins, it is observed expressive increases of planted areas and of productivity on maize culture, which have been higher than the national mean in the last years. These indicators make the state of Tocantins one of the greatest producers of the North region [1], and one of the potential producers of grains of Brazil in a near future [2].

Among the factors that affect the productivity and the quality of the grain, there are the diseases. Recent studies show an increase on the frequency of the fungus *Curvularia lunata* (Wakker) provoking diseases in maize cultivars in China [3]. In maize plantations in Brazil, there are few records of the *Curvularia* Leaf Spot in scientific literature. In the state of Tocantins there is an observed marked increase on the incidence of this disease for many years [4]. In a disease survey on Tocantins realized by [5], the *Curvularia* Leaf Spot was noted together with helmintosporiosis (*H. turcicum*) as more incident.

Nowadays, alternatives for reduction of the use of pesticides are searched, and some works have already been realized aiming at identifying new bioproducts based on medicinal plants with antimicrobial action [6]. Some works have already showed that the addition of essential oils in specific concentrations on the culture environments with phytopathogenic fungi is efficient in the inhibition of development of these organisms [7,8,9,10].

Considering the lack of works developed for the control of *Curvularia* Leaf Spot in the state of Tocantins and in Brazil, studies are necessary in pursuing the disease management. The objective of the present work was to evaluate the fungitoxicity of the essential oil of *C. citratus*, commonly known as lemongrass, upon the phytopathogen *C. lunata,* and also to verify its efficiency on the preventive and curative control of *Curvularia* Leaf Spot on maize.

## 2. Material and Methods

The experiments were performed at the Phytopathology Laboratory of the Gurupi Campus, Federal University of Tocantins (UFT), in the state of Tocantins, Brazil. *C. lunata* was initially isolated from the maize leaves derived from the experimental field of UFT with the diseases’ symptoms, and put in a PDA (Potato, Dextrose, Agar) culture environment. 

### 2.1. Essential Oil Attainment

For the extraction of the oil of *C. citratus* (DC.) Stapf, de Candolle and reclassified by Otto Stapf, the hydrodistillation method was used, utilizing the Clevenger device modified in accordance with the methodology described by [11].

### 2.2. Pathogenicity of the Isolates of Curvularia Lunata to Maize Plants

The pathogenicity of the fungus *C. lunata* was verified through inoculation tests in maize plants. Initially, the seeds of the Traktor (Syngenta^®^) maize were sowed in seven polyethylene vases (3 L capacity) containing substrate, improved with 10 g of commercial fertilizer NPK (5-25-15) in each vase. There were made three holes, with two seeds added in each. The pruning was necessary when the plants reached two pairs of definitive leaves, which was in approximately 15 days, and then the inoculation could be initiated. There was utilized an entirely casualized design with seven treatments and three repetitions. There were different tested concentrations of conidia, being 10^1^, 10^2^, 10^3^, 10^4^, 10^5^, and 10^6^ conidia mL^−1^, the concentrations were adjusted with the aid of a Neubauer chamber. The positive control prepared utilized only distilled water with the same volume utilized for the concentrations of the conidia. For the inoculation there was utilized a manual sprayer (500 mL capacity) containing suspension of conidia of *C. lunata* derived from cultures incubated for 10 days in BOD (Biochemical Oxygen Demand) at 25 °C. The suspensions were applied until the runoff point on the leaves (10 mL). Following this, the vases were kept for 48 h with humid cotton and closed with plastic bags aiming at providing the humid chamber. From 48 h after the inoculation, the vases were left in a shaded place until the appearance of the first leaf symptoms of the *Curvularia* Leaf Spot. When the symptoms were visualized on the inoculated tissue, the fungus was re-isolated and cultivated in PDA environment. Afterwards, the fungus was inoculated again and re-isolated in the PDA environment, aiming at the compliance of all the stages of the Koch’s postulates.

### 2.3. Preparation of the Solutions for Biological Activity

For the preparation of the essential oil solutions sterilized small glass jars with lids were utilized in order to receive the different concentrations. One supply solution was prepared for the higher concentration utilizing a volumetric flask of 10 mL. On sequence, there was added initially the oil and adjusted the concentration of 50 µL mL^−1^ to a volume of 10 mL, next there was added Tween 80 (1 oil: 1 Tween). In laminar flow chamber, the flask was softly agitated and next was inserted the distilled water. After the agitation, there was obtained a homogeneous mixture. Subsequently, there were realized the other dilutions until the concentrations of 2.5 µL mL^−1^, 5 µL mL^−1^ and 7.5 µL mL^−1^ were obtained. As negative and positive control there were prepared the solutions of methyl-thiophanate (2 mg mL^−1^) and sterilized distilled water, respectively.

### 2.4. Phytoxicity of the Essential Oil (Cymbopogon citratus) to Maize Plants

The phytoxicity test was realized in greenhouse conditions. There were utilized six polyethylene vases filled with half commercial substrate and half soil. There was added 10 g of fertilizer NPK (5-25-15) in each vase. On the maize plantation there were utilized Traktor (Syngenta^®^) seeds, sowing two for each hole. After sowing, the vases were irrigated daily until the seedlings growth reached four definitive leaves, or 15 days after plantation. Manual sprayers were used for the application of the treatments. There was utilized an entirely casualized design with six treatments and three repetitions, where different concentrations of the *C. citratus* oil were tested, being: 2.5 µL mL^−1^, 5 µL mL^−1^, 7.5 µL mL^−1^, 10 µL mL^−1^, 50 µL mL^−1^. The positive control was prepared utilizing only distilled water with the same volume utilized for the concentrations of the oil. Each vase was pulverized with 5 mL of the solutions. The evaluation was made according to the phytotoxicity scale adapted from [12] until the tenth day.

### 2.5. In Vitro Inhibiton of Curvularia lunata

Aiming at verifying the effect of essential oil upon the mycelial growth of the phytopatogen, there was different concentrations of *C. citratus* oil were tested, being: 2.5 µL mL^−1^, 5 µL mL^−1^, 7.5 µL mL^−1^, 10 µL mL^−1^ and 50 µL mL^−1^. As negative and positive control there were prepared the solutions of methyl-thiophanate (2 mg mL^−1^) (one for *C. lunata* and the other for *Fusarium* sp.) and sterilized distilled water, respectively. There was tested another pathogen in order to verify the efficiency of the proposed fungicide. There was utilized 100 µL from each concentration, distributed on the surface of the culture environment with the aid of a Drigalski spatula. Next, a mycelium-agar disk with 6 mm diameter was put into the center of the plates. The plates were sealed with PVC (Polyvinyl chloride) film, identified and incubated in BOD at 25 °C for 10 days. The evaluations were realized with digital caliper assessing the mycelial diameter of the fungus tracing two orthogonal axes with each other upon the center of the plates resulting in a arithmetic mean and measured every two days (2, 4, 6, 8 and 10 days). 

### 2.6. Inhibitory Effect to the Germination of Conidia of Curvularia lunata

For the evaluation of the inhibition effect of the *C. citratus* essential oil upon the germination of conidia there was used an entirely casualized design, with seven treatments and three repetitions. The treatments were constituted by the following oil concentrations: 0.625; 1.25; 2.5; 5.0, and 7.5 µL mL^−1^, negative control, with methyl-thiophanate (2 mg mL^−1^) and positive control with distilled/sterilized water. From each oil treatment, there was mixed 100 µL of the oil concentrations and 100 µL of the *C. lunata* conidia concentration (10^4^ conidia mL^−1^). The evaluation was made 12 h after the inoculation in optical microscope.

The plates containing the oil solutions were incubated on a humid chamber (trays) at 27 °C for 12 h. After this period the evaluation was made with approximately 200 conidia where they were counted, and the percentage of germination of conidia of inhibition were calculated, adapted from [13,14].

### 2.7. Chromatographic Analyses of the Essential Oil of Lemongrass

The qualitative and quantitative analyses of the essential oils were realized by chromatography in gas phase, attached to the mass spectrometry CG-MS. The chromatograph utilized was the model Shimadzu GC-210, equipped with mass selective detector model QP2010 Plus, and was operated in these conditions: capillary column of fused silica TRX-5MS (30 m × 0.25 mm × 0.25 μm of film thickness); with the following programming of the temperature on the column: 60–240 °C (3 °C min^−1^); injector temperature: 220 °C; carrier gas helium; and, splitless injection with injected volume of 1 μL of a solution 1:1000 in hexane. For the mass spectrometer (MS), there were utilized the following conditions: impact energy of 70 eV; ion source, and interface temperature: 200 °C. There was injected, at the sample conditions, a homologous series of n-alkanes (C_9_H_20_… C_26_H_54_). The obtained specters were compared with the Nist and Wiley 229 Library’s database and the retention rate calculated to each component was compared to the tabulated, according to [15]. The quantification of essential oils compounds levels were obtained with a gas chromatograph equipped with a flame ionization detector (FIT) using a Shimadzu GC-210 device, in the following methodology: capillary column RTX-5MS (30 m × 0.25 mm × 0.25 μm of film thickness); injector temperature: 220 °C; FIT temperature: 300 °C; with column programming: initial temperature of 60 °C with a heating rate of 3 °C min^−1^ until 240 °C, next, passing to a heating rate of 10 °C min^−1^ until 300 °C, remaining on this temperature for 10 min; drag gas nitrogen (1.18 mL min^−1^); split rate 1:50; column pressure of 115 KPa, and injected volume of 1 μL in hexane.

### 2.8. Curative and Preventive Control of Curvularia Leaf Spot on Maize

There were used plates containing inoculum of seven days of incubation where were added 10 mL of sterilized distilled water for the preparation of the conidia solutions. Utilizing a soft brush, there was realized the detachment of the conidia. This solution was filtered in gauze, and the conidia were counted in the Neubauer chamber. The concentration of 10^4^ conidia mL^−1^ was utilized for the biological activities. Following, the vases were kept for 48 h with humid cotton and closed with a plastic bag in order to provide a humid chamber. 48 h after the inoculation, the vases were left in shaded place until the appearance of the first leaf symptoms of *Curvularia* Leaf Spot. For the evaluation of the severity of the diseases, there was used a notes scale according to [16], where 0 = healthy plant; 1 = less than 1% of leaf area sick; 3 = 1 to 5% of the leaf area sick; 5 = 6 to 25% of the leaf area sick; 7 = 26 to 50% of the leaf area sick; and, 9 = more than 50% of the leaf area sick.

In order to evaluate the preventive effect of the essential oil, an entirely casualized design was used, where different concentrations of the *C. citratus* oil were tested, being: 0.625; 1.25; 2.5; 5.0; and 7.5 mg mL^−1^, and three repetitions. As control, there were utilized water-pulverized plants (positive control) and methyl-thiophanate-pulverized plants at 2 mg mL^−1^ (negative control). From each treatment, 5 mL was sprayed on the plants and one hour after, the plants were inoculated with 5 mL of the conidia solution (10^4^ conidia mL^−1^) of *C. lunata*. The evaluation of the severity of the disease was made every two days after inoculation (5 evaluations on total).

For evaluating the curative effect of the essential oil, an entirely casualized design was used, where different concentrations of the *C. citratus* oil were tested, being: 0.625; 1.25; 2.5; 5.0; and 7.5 µL mL^−1^, and three repetitions. As a control, there were utilized water-pulverized plants (positive control) and methyl-thiophanate-pulverized plants at 2 mg mL^−1^ (negative control). The maize plants were inoculated with 5 mL of the conidia solution (10^4^ conidia mL^−1^) of *C. lunata*, and next the vases were kept for 48 h with humid cotton and closed with plastic bag in order to provide a humid chamber. 48 h after the inoculation, the plants were left in shaded place until the appearance of the first leaf symptoms of *Curvularia* Leaf Spot. From each treatment, 5 mL of the already cited oil concentrations was sprayed on the plants after verification of the appearance of the disease, and the evaluation of severity of the disease was made every two days, being realized a total of five evaluations after the application of the oil solutions. With the obtained results in the evaluations, the Area Under Disease Progress Curve (AUDPC) was calculated, according to [17].

## 3. Results

**Figure 1:**
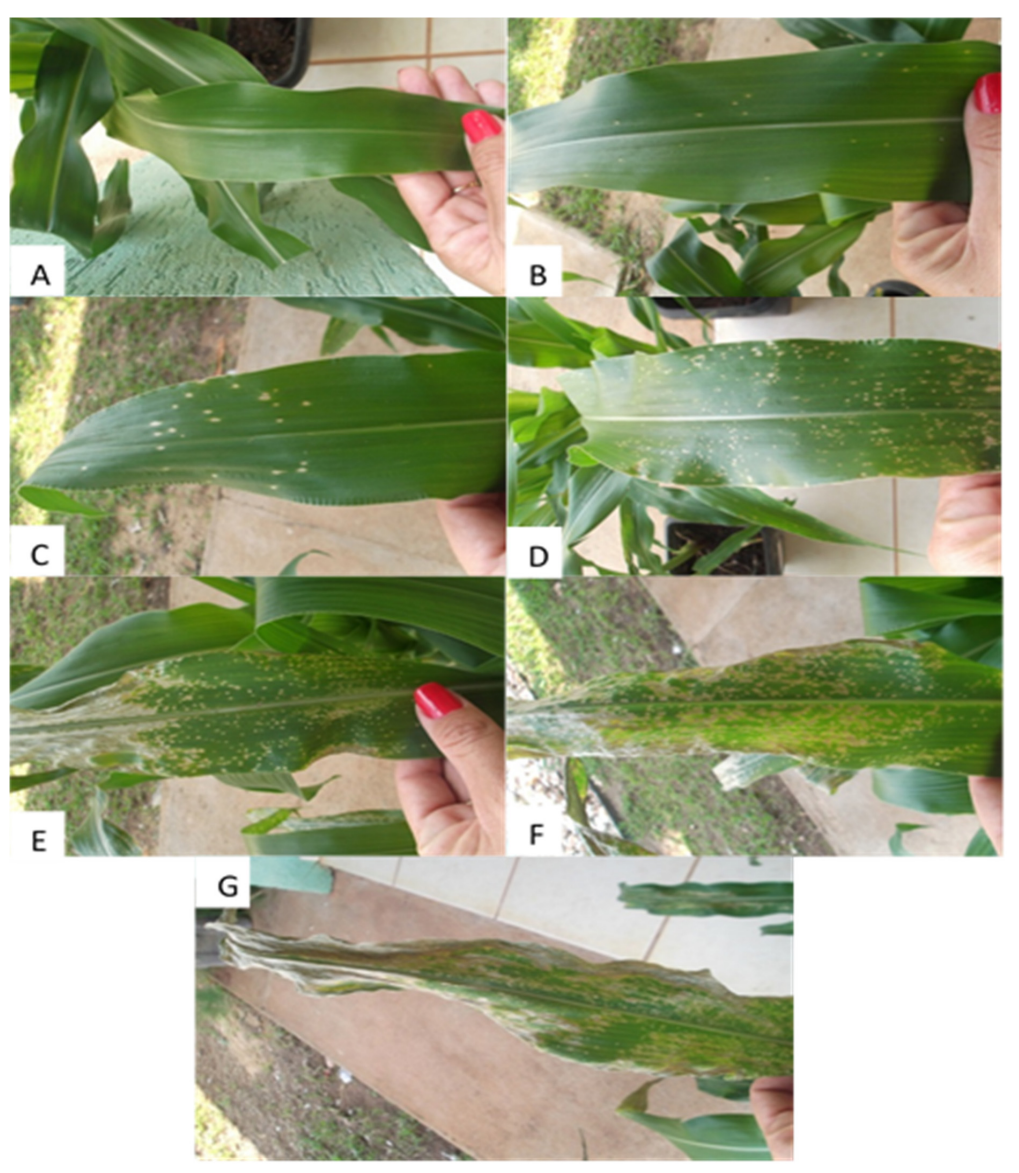
Severity of the *Curvularia* Leaf Spot disease in maize plants, submitted to the inoculation with different conidia concentrations of *Curvularia lunata*. (**A**—Control; **B**—10^1^ conidia mL^−1^; **C**—10^2^ conidia mL^−1^; **D**—10^3^ conidia mL^−1^; **E**—10^4^ conidia mL^−1^; **F**—10^5^ conidia mL^−1^; **G**—10^6^ conidia mL^−1^).

The pathogenicity of the *C. lunata* isolates in maize plants was confirmed, in which typical symptoms were small circular spots on the verified leaves after the incubation period of 48 h later than the inoculation on the plants. However, all the conidia suspensions provoked injuries on the leaves of maize plants (Figure 1). Among the six conidia concentrations prepared (10^1^, 10^2^, 10^3^, 10^4^, 10^5^, and 10^6^ conidia mL^−1^), there was a incidence gradient of leaf spots from the lower concentration to the higher concentration of 10^4^ conidia mL^−1^. The concentration of 10^4^ conidia mL^−1^ was the chosen for the realization of the preventive and curative tests of the next stages realized, due to the better simulation of the severity of the disease verified under favorable conditions to the disease on the field.

**Figure 2:**
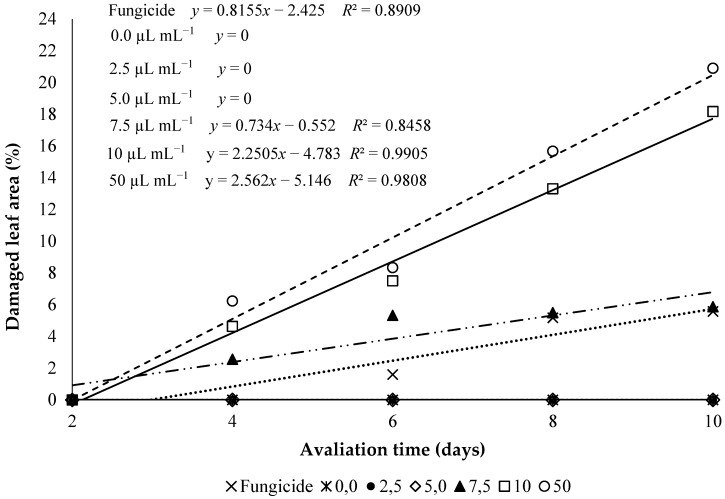
Phytotoxicity (Damaged leaf area x Incubation days) of different *C. citratus* essential oil concentrations in maize plants. Treatments (2.5 µL mL^−1^, 5 µL mL^−1^, 7.5 µL mL^−1^, 10 µL mL^−1^, and 50 µL mL^−1^) positive control (distilled water), negative control—(fungicide—methyl-thiophanate 2 mg mL^−1^).

In the phytotoxicity essay, the non-toxic concentrations were applied in the in vivo tests. Phytotoxicity symptoms appeared after 12 h of the application on leaves. It was verified that at the end of 10 days there was phytotoxicity in the concentrations of 7.5 µL mL^−1^ (5.8%), 10 µL mL^−1^ (18.1%), and 50 µL mL^−1^ (20.9%), thus showing that cannot be utilized in tests of control of the disease in the plant, provoking leaf necrosis, followed by posterior wilt. Yet the concentrations 2.5 µL mL^−1^ and 5.0 µL mL^−1^ did not provoke phytotoxicity along the evaluation (Figure 2).

In the effect of the in vitro inhibition of the *C. citratus* essential oil upon *C. lunata*, it was verified the growth of the phytopathogen in all the tested concentrations (Table 1). Regarding the concentration of 50 µL mL^−1^, the growth was partially inhibited, with values lower than the control values.

Compared the mycelial growth means of the pathogen on the 10th day of incubation, it was verified that the *C. citratus* essential oil did not provide an inhibitory effect among the concentrations of 2.5 µL mL^−1^ (Table 1). Thus, on these concentrations, the phytopathogen mycelium grew normally along the days, and only in the concentrations of 50 µL mL^−1^ (C5) was the partial inhibitory effect observed (Table 1).

For the *C. lunata* phytopathogen, the negative control (methyl-thiophanate) did not present a relevant difference from the positive control (water), even with the utilization of the concentration above the recommended dosage. Thus, not efficiency in the mycelial growth inhibition of this pathogen in vitro was observed. On the other side, it was verified mycelial inhibition of the *Fusarium* genre when submitted to the same fungicide (negative control 2), and in the same concentration. 

**Figure 3:**
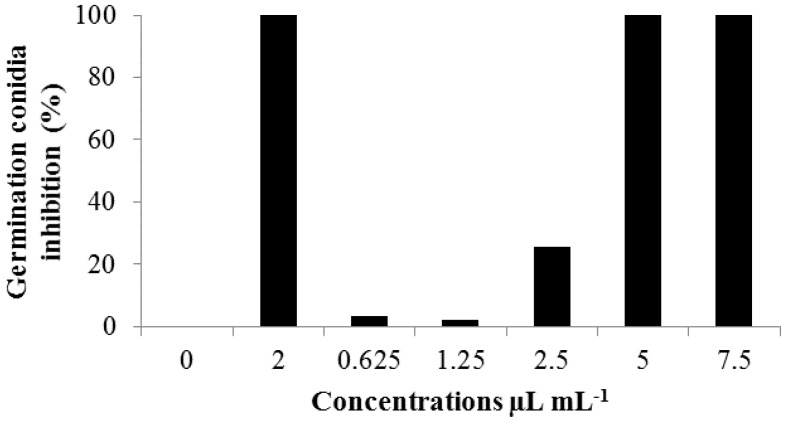
Conidia germination inhibition of *Curvularia lunata* under crescent dosages of *C. citratus* (*Cymbopogon citratus*) essential oil. Treatments: 0 (0.0 µL mL^−1^), 2 (Negative control, with methyl-thiophanate 2 mg mL^−1^). Concentrations of *C. citratus* essential oil = 0.625 µL mL^−1^; 1.25 µL mL^−1^; 2.5 µL mL^−1^; 5 µL mL^−1^ and 7.5 µL mL^−1^).

On the essay of *C. lunata* conidia germination inhibition, it was verified that it had 100% of conidia germination on the positive control (methyl-thiophanate). The concentrations of 5.0 and 7.5 µL mL^−1^ of the *C. citratus* oil also totally inhibited the conidia germination (Figure 3). Yet the concentrations 0.625; 1.25 and 2.5 µL mL^−1^ had a low inhibition percentage of 3.3%, 2.3%, and 25%, respectively, indicating oil activity upon the conidia, even in the lower concentrations.

It can be observed (Table 2) that the majoritarian chemical components present on the essential oil of *Cymbopogon citratus* leaves were Geranial (41.46%) and Neral (32.43%).

In the preventive and curative effects, the utilized concentrations were different from the tested in vitro (fungitoxicity) and in vivo (phytotoxicity), because from the concentration 7.5 µL mL^−1^, the plants presented phytotoxicity symptoms. Therefore, there were tested concentrations below the utilized in in vitro tests.

**Figure 4:**
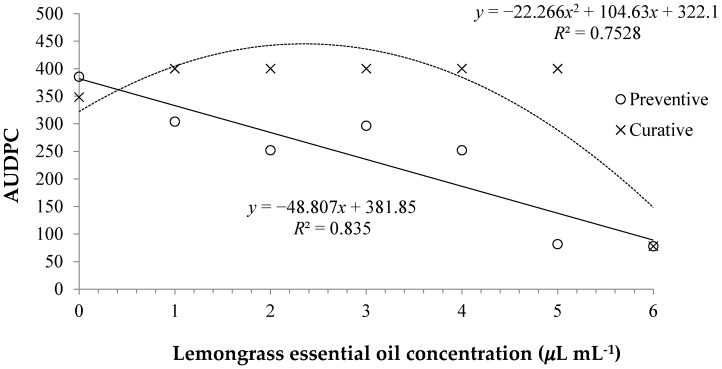
Area Under Disease Progress Curve (AUDPC) for preventive and curative in vivo under positive control with water (0 = 0.0 µL mL^−1^) and negative control with methyl-thiophanate (6 = 2.0 mg mL^−1^), with five different concentrations (1 = 0.625 µL mL^−1^, 2 = 1.25 µL mL^−1^, 3 = 2.5 µL mL^−1^, 4 = 5 µL mL^−1^, and 5 = 7.5 µL mL^−1^) of the *Cymbopogon citratus* essential oil.

For the preventive effect of the *C. citratus* essential oil under progress of the *Curvularia* Leaf Spot disease (Figure 4), it was observed a greater Area Under Disease Progress Curve (AUDPC) in the positive control (Distilled water), in relation to the other treatments. For the plants treated with the fungicide methyl-thiophanate (6) there was reduction on the disease symptoms in relation to most of the utilized treatments. However, in the oil concentration 7.5 µL mL^−1^ a similar effect to the control with methyl-thiophanate was verified. The other concentrations had similar AUDPC.

Regarding the curative control (Figure 4), it was observed that the application of *C. citratus* essential oil did not provide the expected effect and there was no control of the disease on the symptomatic plants and the treatments AUDPC was similar to the control. Yet the treatment with methyl-thiophanate was also efficient on the curative control of the disease.

## 4. Discussion

The phytoxicity on maize plants has as a principal symptom the appearance of necrosis on the leaves, specifically on the regions in which the solution accumulates, like borders and leaf veins, causing a loss of the green leaf area [18]. Studies realized by [19], show that the concentration of 1% of the *C. citratus* oil showed presented low toxicity (≤25%), since the plants presented only a light necrosis on the leaves.

The *C. citratus* essential oil has as majoritarian component the Citral, with 69.31% (Geranial + Neral) and the Myrcene, with 23.77%, both being monoterpenes, according to [20] studies, showing that both the oil and its majoritarian component, the Citral, acted on the mycelial inhibiton of the phytopathogens *Fusarium oxysporum cubense*, *Colletotrichum gloeosporioides*, *Bipolaris sp.* and *Alternaria alternata*.

When observing the fungistatic action in vitro, [10] noted that the lemongrass (*C. citratus*) essential oil inhibited the development of the fungi *Didymella*, *bryoniae*, *pyricularia grisea*, *Rizoctonia solani* and *Sclerotium rolfsii*, demonstrating the high potential for the alternative control of these pathogens. Studies realized by [21] obtained the total inhibition of the mycelial growth and of the germination of spores from *Colletotrichum gloeosporioides* utilizing the *C. citratus* essential oil from the concentration of 1 μL mL^−1^.

The chemical composition of the *C. citratus* essential oil is described in many studies. Elevated levels of Geranial and Neral in the chemical composition of lemongrass were observed by [22], with a mean of approximately 51% and 36%, respectively. The authors [23] also observed similar percentages for Geranial (46.3%) and Neral (31.28%). Yet [24] found levels of 43.6% for Neral and 34% for Geranial, values that are different to the ones found on this work, where the highest percentage was of Neral. It is known that many factors, such as extraction method, access and conditions of cultivation may have an influence on the levels of the essential oil components. That can explain the differences found in the present study.

There is no record in the scientific literature about the preventive and curative effect in vivo of any essential oil upon the *Curvularia* Leaf Spot disease. Nevertheless, [25] when studying another pathosystem, it demonstrated good results for the alternative control. Five treatments were tested with lemongrass and other essential oils against the helmintosporiosis of Tanzania grass (*Panicum maximum* Jacq.) in preventive and curative effect.

It was verified by [19], on the control of the phytopathogen *Colletotrichum graminicola*, that the lemongrass essential oil stood out as the most fungitoxic, since that occurred inhibition of 100% of fungus mycelial growth from the concentration (0.50 μL mL^−1^). The fact that the *C. citratus* essential oil was not efficient on the in vitro inhibition test, but effective on the conidia germination and preventive control, demonstrates that it is very important to realize essays in vivo, but is also necessary in future works, in order to enlighten the mechanism of action of essential oils upon the plant diseases.

## 5. Conclusions

The *Curvularia lunata* isolate is pathogenic to the maize plants on the tested concentrations. For the phytotoxicity essay the concentration above 7.5 µL mL^−1^ was phytotoxic to the plants, marking off the maximum concentration to be utilized. The *C. lunata* phytopathogen showed itself resistant to the methyl-thiophanate in a concentration applied above the recommended in in vitro tests. In the in vitro control the tested dosages of *C. citratus* essential oil presented promising results and can be a great alternative on the management of this disease, however the curative application was not efficient.

## Figures and Tables

**Table 1:** Mean mycelial diameter (mm) of *Curvularia lunata* under different concentrations of the *C. citratus* essential oil.

Treatments	Avaliation Time (Incubation Days)					Regression Equation	R^2^
	2	4	6	8	10		
PC	26.21	44.69	59.44	68.96	80.56	y = 13.297x + 16.081	0.98
NC ^a^	25.27	42.51	59.44	67.4	78.11	y = 13.057x + 15.375	0.97
NC ^b^	8.59	9.14	9.74	9.94	10.51	y = 0.464x + 8.192	0.98
C_1_	25.06	40.90	55.12	69.72	82.89	y = 14.448x + 11.394	0.99
C_2_	24.36	40.22	53.31	69.05	82.62	y = 14.535x + 10.307	0.99
C_3_	21.4	36.27	52.35	68.29	82.36	y = 15.394x + 5.952	0.99
C_4_	19.97	36.08	51.96	66.74	81.35	y = 15.342x + 5.194	0.99
C_5_	18.09	35.54	50.74	66.61	77.37	y = 14.963x + 4.781	0.99

PC: Positive control (distilled water), NC ^a^: Negative control 1 (methyl-thiophanate under *Curvularia*), NC ^b^: Negative control 2 (methyl-thiophanate under *Fusarium sp.*), C_1_–C_5_: Concentrations in µL mL^−1^ (C_1_ = 2.5 µL mL^−1^, C_2_ = 5 µL mL^−1^, C_3_ = 7.5 µL mL^−1^, C_4_ = 10 µL mL^−1^ and C_5_= 50 µL mL^−1^).

**Table 2:** Chemical components of the *Cymbopogon citratus* essential oil identified by mass spectrometry (CG/MS) and its respective levels expressed in percentage.

Lemongrass Essential Oil			
Constituints	RT	IR	(%)
Mircene	7.742	986	9.73
(Z)-β-oxime	9.344	1020	0.32
(E)-β-oxime	9.753	1029	0.16
Linalool	11.845	1074	1.64
Neral	17.885	1209	32.43
Geraniol	18.375	1220	4.52
Geranial	19.233	1239	41.46
2-undecanone	20.099	1359	0.35
Geranyl acetate	23.737	1443	0.42
E-Caryofylene	31.997	1641	0.17
Anothers	-	-	8.8
Total	-	-	100

RT = retention time; IR = calculated retention rate.
